# Functions and properties of nuclear lncRNAs—from systematically mapping the interactomes of lncRNAs

**DOI:** 10.1186/s12929-020-00640-3

**Published:** 2020-03-17

**Authors:** Chia-Yu Guh, Yu-Hung Hsieh, Hsueh-Ping Chu

**Affiliations:** Institute of Molecular and Cellular Biology, National Taiwan University, No. 1 Sec. 4 Roosevelt Road, Taipei, Taiwan, Republic of China

**Keywords:** Long non-coding RNA, RNA-interactome, Epigenetics, Chromatin, Nuclear architecture, R-loops, Phase separation

## Abstract

Protein and DNA have been considered as the major components of chromatin. But beyond that, an increasing number of studies show that RNA occupies a large amount of chromatin and acts as a regulator of nuclear architecture. A significant fraction of long non-coding RNAs (lncRNAs) prefers to stay in the nucleus and cooperate with protein complexes to modulate epigenetic regulation, phase separation, compartment formation, and nuclear organization. An RNA strand also can invade into double-stranded DNA to form RNA:DNA hybrids (R-loops) in living cells, contributing to the regulation of gene expression and genomic instability. In this review, we discuss how nuclear lncRNAs orchestrate cellular processes through their interactions with proteins and DNA and summarize the recent genome-wide techniques to study the functions of lncRNAs by revealing their interactomes in vivo.

## Introduction

Over the past decade, intensive studies have demonstrated that long non-coding RNAs (lncRNAs) are key regulators in embryonic development [1], DNA damage responses [2], and human diseases such as neuronal disorders [3], heart dysfunction [4], and cancers [5, 6]. LncRNAs can act as effectors to direct biological processes. Unlike DNA, RNA is more mobile, flexible, able to self-fold into a distinct structure and interact with DNA or RNA by base pairing. Revealing the molecular mechanisms by which lncRNAs regulate those biological functions relies on the study of their interaction between DNA and proteins. Several methodologies have been developed to systematically identify RNA interacting chromatin, RNA interacting proteins and RNA-RNA interactions in vivo, thus uncovering the network of protein-RNA-DNA (Table 1).

**Table 1:** In vivo mapping RNA interactomes

Method	Cross-linking	Concept	Advances	Identifier	Ref
**RNA-chromatin**					
ChIRP-seq	Glutaraldehyde	Use biotinylated antisense oligos to pull down a targeted RNA with its associated DNA.	Robust elution by RNase A and RNase H.	DNA that is associated with a specific RNA	[7]
CHART-seq	Formaldehyde	Use biotinylated antisense oligos to pull down a targeted RNA with its associated DNA.	Apply RNase H to specifically elute RNA mediated interacting chromatin.	DNA that is associated with a specific RNA	[8, 9]
CHIRT-seq	Glutaraldehyde	A hybrid method of ChIRP and CHART.	Combination of glutaraldehyde fixation and RNase H elution.	DNA that is associated with a specific RNA	[10]
MARGI-seq	Formaldehyde	Use a linker to ligate RNA and DNA in proximity to form of RNA-linker-DNA.	Reveal all interactions between DNA and RNA.	All RNA-DNA contacted sequences	[11]
ChAR-seq	Formaldehyde	Use a linker to ligate RNA and DNA in proximity to form of RNA-linker-DNA.	Reveal all interactions between DNA and RNA.	All RNA-DNA contacted sequences	[12]
GRID-seq	Formaldehyde and disuccinimidyl glutarate (DSG)	Use a linker to ligate RNA and DNA in proximity to form of RNA-linker-DNA.	Strong crosslinking to reveal long-range interaction between DNA and RNA.	All RNA-DNA contacted sequences	[13]
HiChIRP-seq	Glutaraldehyde	Combine ChIRP and Hi-C. Use CLICK chemistry to conjugate a biotin for subsequent contact enrichment.	Characterize a specific RNA that involves in chromosomal interaction.	Chromosome conformation at a specific RNA associated sites	[14]
**RNA-proteins**					
CLIP-seq	UV irradiation (254 nm)	Pull down RNA-protein complexes by immunoprecipitation and perform reverse transcription.	Identify all RNAs that interact with a targeted protein.	RNA that binds to a specific protein	[15]
iCLIP-seq	UV irradiation (254 nm)	Pull down RNA-protein complexes by immunoprecipitation and perform reverse transcription.	A random barcode to mark individual cDNA molecules to solve the problems of PCR duplicates.	RNA that binds to a specific protein	[16]
PAR-CLIP-seq	Incorporate 4-thiouridine (4-SU) and 6-thioguanosine (6-SG) into nascent RNA. UV (365 nm)	Builds on CLIP. Incorporation of 4-SU or 6-SG results in U to C and G to A mutations respectively that allows mutational analysis to identify cross-linked sites.	Use 4-SU or 6-SG incorporation to increase the crosslinking efficiency.	RNA that binds to a specific protein	[17]
RAP-MS	UV irradiation (254 nm)	Use biotinylated antisense RNA probes to capture a specific RNA.	Identify direct RNA interacting proteins.	Proteins that bind to a specific RNA	[18]
ChIRP-MS	Formaldehyde	Use biotinylated antisense DNA probes to capture a specific RNA.	Identify direct and indirect RNA interacting proteins.	Proteins that bind to a specific RNA	[19]
iDRiP-MS	UV irradiation (254 nm)	Use biotinylated antisense DNA probes to capture a specific RNA.	Identify direct RNA interacting proteins.	Proteins that bind to a specific RNA	[20] [10]
RBR-ID	UV (312 nm) + 4-thiouridine (4-SU)	Comparison of 4-SU and non-4-SU treatments, an RNA-crosslinked peptide has a different mass.	Identify all proteins bound to RNAs.	All RNA binding proteins	[21]
**RNA structure/ RNA-RNA interactions**					
FragSeq	N/A	RNA is digested by P1 endonuclease. Nuclease probing.	Map P1 endonuclease digestion sites.	In vitro RNA structure	[22]
PARS	N/A	RNA is digested by RNase V1 or S1 to determine double stranded or single stranded regions. Nuclease probing.	Map RNase V1 or S1 digestion sites.	In vitro RNA structure	[23]
SHAPE-seq	Covalently modify RNA molecules in vitro.	SHAPE reagents (1 M7, NAI-N_3_) modify RNAs.	Single nucleotide resolution; each RNA in the experiment is bar-coded.	In vitro RNA structure	[24] [25, 26]
icSHAPE-seq	Covalently modify RNA molecules in vivo	SHAPE reagent (NAI-N_3_). Copper-free click chemistry, a biotin moiety is selectively and efficiently added to NAI-N_3_-modified RNA.	Identify In vivo RNA structure.	In vivo RNA structure	[27]
DMS-seq (Structure-seq)	Covalently modify RNA molecules	Dimethyl sulphate (DMS) methylates the base-pairing faces of A and C of RNA in loops.	Nucleotide resolution. Map RNA structure in vivo.	In vivo RNA structure	[28]
COMRADES	Psoralen + UV irradiation (365 nm)	Pull down a specific RNA using biotinylated DNA oligos and perform proximity ligation.	Reveal RNA structures and interactions of a specific RNA in vivo.	In vivo RNA structures and interactions of a targeted RNA	[29]
CLASH	UV irradiation (254 nm)	Immunoprecipitation to enrich a specific RNA binding protein and perform linker ligation.	Find mRNA target sequences for miRNA.	RNA hybrids bound by a specific RNA-binding protein	[30] [31]
hiCLIP	UV irradiation (254 nm)	Immunoprecipitate RNA-protein complexes by using antibodies against a specific RNA-binding protein and ligate RNA duplexes in proximity.	Reveal RNA duplexes bound to a specific protein.	RNA duplexes bound by a specific RNA-binding protein	[32]
MARIO	Formaldehyde and EthylGlycol bis	Apply a biotinylated linker to ligate two RNA fragments in proximity.	Reveal all RNA-RNA interactions.	All RNA fragments in proximity	[33]
PARIS	Psoralen + UV irradiation (365 nm)	Purify RNA-duplexes by 2D gel and ligate two RNA fragments in proximity	Reveal all RNA-RNA interactions	All RNA duplexes	[34]
SPLASH	Psoralen + UV irradiation (365 nm)	Use biotinylated psoralen to crosslink RNA and perform proximity ligation.	Reveal all RNA-RNA interactions.	All RNA-RNA hybrids	[35]
**RNA-DNA hybrids (R-loops)**					
DRIP-seq	N/A	Pull down RNA/DNA hybrids using S9.6 antibody that specifically recognizes RNA/DNA hybrids.	Reveal DNA-RNA hybrids.	DNA that forms hybrids with RNA	[36, 37]
bisDRIP-seq	Bisulfite modification	Use bisulfite to convert cytosine residues into uracil residues within genomic DNA regions that contain single-stranded DNA. Enrich DNA/RNA hybrids by S9.6 IP.	Define the boundaries of the R-loop, high resolution.	Single-stranded DNA of R loops	[38]
R-ChIP	Formaldehyde	Use catalytic-dead RNase H to capture R loops, similar to ChIP.	Reveal DNA-RNA hybrids, high resolution.	RNase H target sites, R loops	[39]
DRIPc-seq	N/A	Builds on DRIP. Sequence RNA of DNA-RNA hybrids.	Reveal DNA-RNA hybrids, high resolution.	RNA of R loops	[40]

### Chromatin associated RNAs

**Fig. 1:**
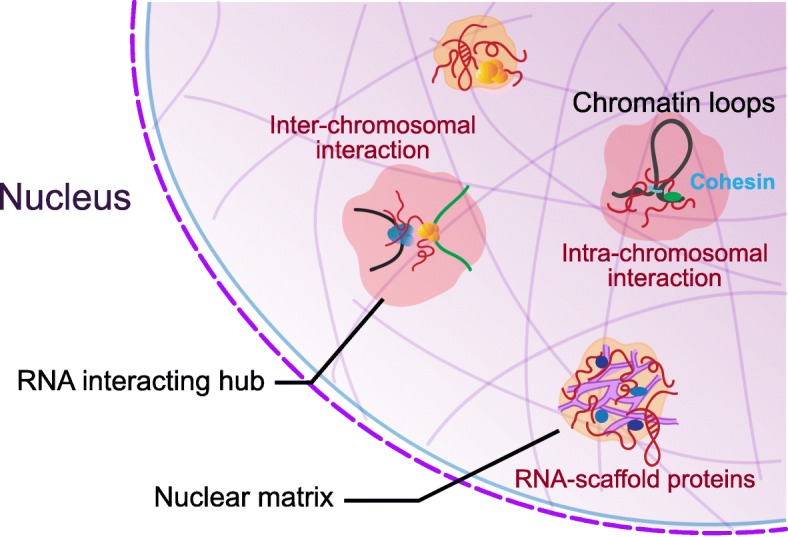
lncRNA-interacting hub in the nucleus. LncRNAs serve as scaffolds for protein complexes and bring two or several distant DNA loci together. RNA involves the maintenance of nuclear architecture, facilitates chromatin looping, as well as directs the inter- or intra-chromosomal interactions

The first study showed that RNA constitutes a significant fraction of chromatin date back over 50 years ago [41, 42]. Later studies provide evidence to support the idea that a nuclear matrix is made of insoluble proteins and RNA in an interphase nucleus [43]. Treatment of RNase A leads to clumping of chromatin onto nuclear lamina and nucleolus [44, 45], indicating an indispensable role of RNA in the nuclear architecture. However, identifying a specific RNA contributes to a specific phenotype in the nucleus had facing technical challenges before the 1990s. Recently, the burst of studies utilized next-generation sequencing to characterize the interactions of lncRNA and chromatin. While using lncRNA ablation, accumulating evidence has suggested that RNA-protein complexes conduct various functions such as the formation of chromatin compartment, gene regulation, and inter- or intra-chromosomal interactions [44, 45] (Fig. 1).

It has been reported that the CCCTC-binding transcription factor (CTCF), which promotes chromatin loop formation, binds to thousands of RNAs [46]. Depletion of steroid receptor *RNA* activator (SRA) noncoding RNA that is associated with CTCF, reduced CTCF-mediated insulator activity [47] at the *IGF2/H19* imprinting control region and increased *IGF2* expression. It was also reported that a lncRNA, Jpx, activates Xist RNA expression by evicting CTCF from binding to *Xist* promoter [48]. These results imply that lncRNAs in the nucleus cooperate with RNA-binding proteins to regulate the gene expression, perhaps through modulating the chromatin conformation.

It was proposed that lncRNA transcription guides genome organization [49]. For example, Oakes et al. demonstrated that inhibition of rRNA transcription leads to nucleolus disassembly [50]. Induction of the transcription of rRNA genes that are inserted into new chromosomal locations can generate nucleolus-like structures [51]. These results suggest that rRNA transcription could be responsible for the nucleolus organization. Lately, Nozawa et al. had suggested that chromatin-associated RNAs can form a chromatin mesh with scaffold attachment factor A (SAF-A), also known as heterogeneous ribonucleoprotein U (HNRNP-U) [52]. They showed that SAF-A oligomerization, which is dependent on ATP and RNA, drives chromatin decompaction, whereas its monomerization compacts large-scale chromatin organization. The global change of large-scale chromatin by depletion of SAF-A did not alter dramatically gene expression [52], nevertheless, it resulted in excessive DNA damages and genomic instability [52].

### Xist RNA regulates gene silencing and chromatin conformation

**Fig. 2:**
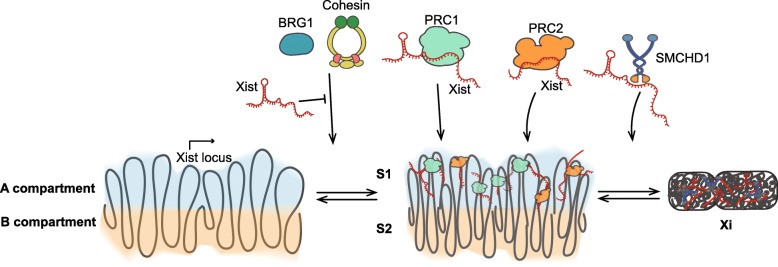
Xist RNA tethers epigenetic regulators and contributes 3D conformation of the X chromosome. Xist RNA recruits repressive complexes such as polycomb complexes PRC1 and PRC2 to the inactive X chromosome (Xi) to facilitate heterochromatin formation, thus leading to gene silencing. On the other hand, Xist binds to BRG1 and cohesin and repels them from the Xi to prevent TAD (topologically associated domain) formation. Xist mediates the Xi folding in 3D space by tethering SMCHD1, which facilitates the merge of chromatin compartments. A/B compartments are first fused into “S1” and “S2” compartments, after SMCHD1 recruitment, further merging to compact Xi structure

Xist RNA is one of the well-studied lncRNAs involved in epigenetic regulation and gene silencing. It is a 17-kb lncRNA that is expressed from the inactive X-chromosome (Xi) [53], coating the entire X chromosome to repress gene expression through its ability to recruit repressive complexes such as polycomb complexes PRC1 and PRC2 [54–56]. In addition, Xist plays an important role in three-dimensional (3D) conformation and maintains the heterochromatin structure in Xi (Fig. 2). When Xist was depleted, topologically associated domains (TADs) were restored *in cis* and the Xi (inactive X chromosome) became Xa (active X chromosome) like conformation [20]. Lee et al. also showed that Xist repels positive chromatin factors such as BRG1 (also known as SMARCA4) and cohesin from the Xi [20, 57] to prevent the formation of TADs and chromatin superloops. In a higher order of chromatin structure, mammalian chromosomes are organized into alternating “A/B compartments” in 3D conformation. Spatial compartments usually possess chromatin of similar states, with A compartments being actively transcribed and gene-rich, and B compartments being transcriptionally inactive and gene-poor [58, 59]. Remarkably, ablating SMCHD1 (known as an architectural protein) displayed another layer of compartments--S1/S2 [60] in Xi. Their results indicate that SMCDH1 binds to Xist and facilitates the folding of the S1/S2 compartments into a compartment-less (more compact) structure in Xi (Fig. 2). Altogether, robust evidence has shown that a lncRNA can regulate gene expression and chromatin 3D conformation through its abilities to recruit epigenetic factors, or to act as a decoy or a scaffold for protein complexes.

### Enhancer RNAs promote chromatin looping

Enhancer RNAs were first discovered by two early studies through the genome-wide sequencing such as RNA-seq or ChIP-seq [61, 62]. They demonstrated that enhancers were occupied by RNA polymerase II (RNAP II) and transcribed into a class of ncRNAs termed enhancer RNAs (eRNAs). The epigenetic features of enhancers consist of histone 3 lysine 4 monomethylation (H3K4me1), histone 3 lysine 27 acetylation (H3K27ac), histone variants (H2AZ, H3.3), co-activators (mediator complex), and an open-chromatin architecture (DNase I hypersensitivity) [63]. The expression of enhancer RNAs could be regulated by various stimuli such as estrogen (ER) or androgen (AR) [64–66]. It is generally believed that eRNAs are unstable, transcribed quickly after induction, and degraded rapidly [62]. Several lines of evidence suggest that eRNAs can promote enhancer-promoter looping or facilitate RNA pol II loading, thus upregulating their target genes [65, 67]. For example, the gonadotropin hormone α-subunit gene is regulated by the eRNA in a cell-type-specific manner [68, 69]. The depletion of the eRNA results in a loss of interactions of the enhancers and promoters of the gonadotropin gene [68]. These results further support that lncRNAs enable to direct chromatin looping.

### lncRNAs mediate interchromosomal interactions

Homologous chromosome pairing rarely occurs in somatic cells under normal growth conditions with only a few exceptions. Transvection was first observed in 1908 in *Drosophila*, where homologous chromosomes were closely paired in somatic nuclei [70]. It is an epigenetic phenomenon that can lead to either gene activation or repression. X chromosome pairing is one of the best-known examples of somatic homologous pairing in mammals [71]. Tsix, a lncRNA*,* is highly expressed in undifferentiated ES cells, and antagonizes Xist action. During ES differentiation in female cells, transient homologous pairing occurs, and the Xist RNA expression increases to initiate chromosome-wide silencing. It was reported that Tsix RNA mediates the homologous pairing between two X chromosomes, thus breaking epigenetic symmetry between the two X chromosomes, as well as driving the random choices for the selection of inactive or active X chromosomes [72, 73]. Moreover, another lncRNA derived from the ends of sex chromosomes, dubbed PAR-TERRA (telomeric repeat-containing RNA), facilitates the pairing by clustering the ends of the sex chromosomes, and creates a hub to constrain the DNA loci in 3D space [10]. Not limited to X chromosomes, several studies indicated that somatic allelic pairing also occurs in a number of autosomal loci, such as *Oct4* and various cytokine genes [74–77]. Although how interchromosomal pairing impacts gene expression and epigenetic regulation remains elusive, it has been proposed that the alignment of the two homologous alleles could allow bi-allelically bound transcription factors to redistribute onto one allele to achieve their lowest free energy state (the aggregated state) [72, 78, 79], thus inducing the transition from biallelic to monoallelic expression.

### Methods for RNA-chromatin interactions

**Fig. 3:**
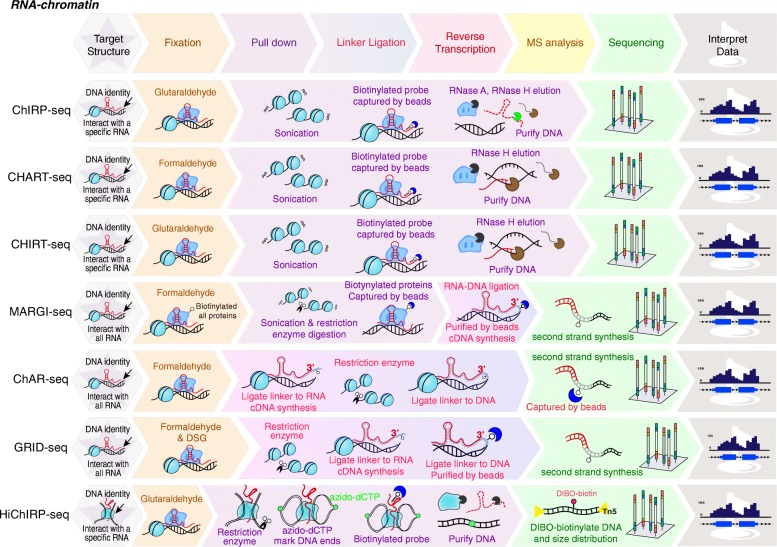
Mapping RNA-chromatin interactions. In ChIRP-seq, CHART-seq, and CHIRT-seq, RNA associated chromatin complexes are crosslinked by formaldehyde or glutaraldehyde, captured by antisense oligos that target a specific lncRNA, and DNA fragments that are associated with RNA-protein complex are sequenced. For all-to-all interactions (MARGI-seq, ChAR-seq, and GRID-seq), a linker is ligated to connect DNA and RNA. DNA-RNA chimeras are sequenced. HiChIRP-seq combines Hi-C and ChIRP to identify the interacting chromatin (inter- and intra-chromatin interactions) of a specific lncRNA

To map the RNA-associated chromatin in vivo, Chu et al. and Simon et al. utilized biotinylated antisense DNA oligo probes to capture a specific RNA that is associated with chromatin [7, 8] (Table 1 and Fig. 3), named ChIRP-seq (Chromatin isolation by RNA purification) and CHART-seq (Capture Hybridization Analysis of RNA Targets) respectively. They first fixed cells with either glutaraldehyde (ChIRP) or formaldehyde (CHART) and sheared chromatin into small pieces by physical sonication. To minimize the noises caused by the non-specific interactions of DNA probes and chromatin DNA or proteins, CHART includes an RNase H step to elute the RNA-chromatin complexes. This is to ensure that only lncRNAs-complexes that are targeted by DNA probes will be eluted by RNase H, which specifically degrades RNA of RNA-DNA hybrids. Later, a hybrid method was developed, called CHIRT-seq, which combines glutaraldehyde fixation and RNase H elution [10] to identify genomic binding sites for TERRA RNA. Accumulating studies have used ChIRP-seq to determine the genomic bindings of many lncRNAs, including NEAT1, MALAT1, HOTAIR and MEG3*,* TERC, LTR ERV-9 [80, 81]. CHART-seq had also successfully identified Xist, NEAT1, and MALAT1 genomic binding sites [8, 9].

Recently, several methods were developed to reveal all interactions between RNA and DNA in the nucleus, including MARGI-seq (Mapping RNA-genome interactions), ChAR-seq (Chromatin-Associated RNA sequencing), and GRID-seq (Global RNA Interactions with DNA) [11–13]. The idea of these methods is to ligate chromatin-associated RNAs with their target genomic sequences by proximity ligation using a linker, thus forming RNA-DNA chimeric fragments. These techniques revealed hundreds of chromatin-associated RNAs including previously known lncRNAs *and* a large set of non-coding RNAs that are bound to active promoters, enhancers, and super-enhancers in a tissue-specific manner [11–13]. All-to-all mapping ideally can discover all interactions of RNA-chromatin, however, the coverage depends on the ligation efficacy, the distances between RNA and genomic DNA, as well as the abundance of RNAs.

As discussed previously, long non-coding RNAs may serve as scaffolds to bring two or several distant DNA loci into spatial proximity. To understand how a specific RNA interacts with chromatin in a 3D space, Mumbach et al. developed a method, named HiChIRP, which combines Hi-C and ChIRP [14]. They incorporated azido-CTP into chromatin contacts, captured RNA-chromatin using biotinylated probes, conducted the copper-free dibenzocyclooctyne (DIBO) ‘CLICK’ chemistry to covalently conjugate a biotin for subsequent contacts, and sequenced the DNA contacts. They performed HiChIRP on 7SK small nuclear RNA (snRNA), telomerase RNA component (TERC) and lincRNA-EPS [14]. They found that thousands of loops were enriched on 7SK HiChIRP, and some of them in promoters and active regulatory elements. They also showed that TERC is not only associated with loops that formed between telomeres but also with enhancer-promoter loops at some oncogenes, implying a role of TERC outside of telomeres. Therefore, their results provide insights into how lncRNAs mediate inter- or intra-chromatin looping.

### Methods for RNA-protein interactions

**Fig. 4:**
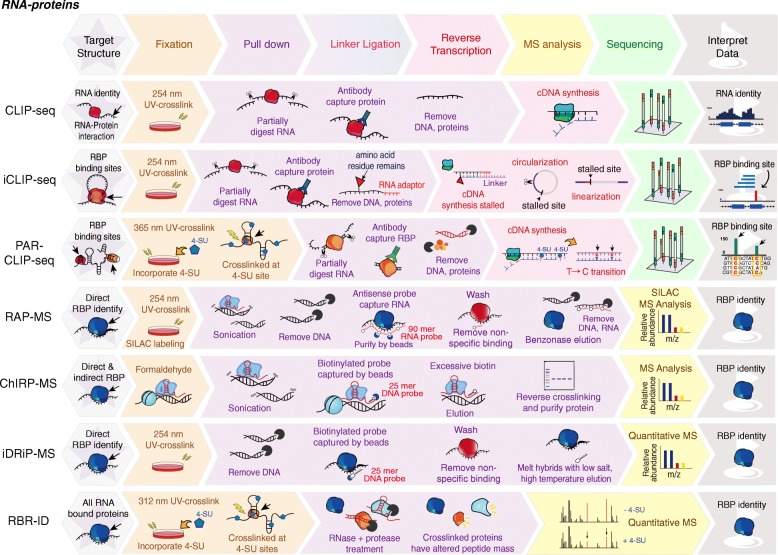
Mapping RNA-protein interactions. In CLIP-seq, iCLIP-seq, and PAR-CLIP-seq**,** RNA associated chromatin complexes are crosslinked by UV light, pulled down using antibodies against a specific protein of interest, and the captured RNA are sequenced. In PAR-MS, ChIRP-MS, and iDRiP-MS, RNA-protein complexes crosslinked by UV light or formaldehyde, captured by antisense oligos, and the pulled-down proteins are identified by mass spectrometry. RBR-ID utilizes protein-RNA photocrosslinking and quantitative MS to identify all proteins that interact with RNA

RNA binding proteins (RBPs) are associated with a large number of human disorders, such as autoimmune and neurologic diseases [82–84]. Remarkable examples include FMRP in the Fragile-X mental retardation protein [83], the neuron-specific Nova and Hu proteins in the paraneoplastic neurologic degenerations [84] and the small nuclear ribonucleoproteins (snRNPs) in systemic lupus erythematosus [82]. To identify RNAs that interact with these proteins in vivo, Ule et al. combined UV cross-linking with immunoprecipitation (CLIP) to pull down RNA-protein complexes [15] (Table 1 and Fig. 4), and the captured RNAs are sequenced. Because CLIP relies on reverse transcription to pass over residual amino acids that covalently attach to the RNA at the cross-link site, cDNAs tend to prematurely truncate immediately before the cross-link nucleotide [15]. Later on, they resolved this problem by PCR amplification of truncated cDNAs via self-circularization of cDNAs, and developed individual-nucleotide resolution UV cross-linking and immunoprecipitation (iCLIP) to precisely map protein–RNA interactions [16]. Due to the fact of low efficiency of RNA-protein crosslinking by UV 254 nm in CLIP, Tuchi et al. developed a method, named PAR-CLIP (photoactivatable ribonucleoside-enhanced crosslinking and immunoprecipitation) [17], to improve the crosslinking efficiency by incorporating 4-thiouridine (4SU) into nascent RNA transcripts in living cells. The cells were then irradiated by UV 365 nm to induce efficient crosslinking of photoreactive nucleoside (4SU)-labeled RNAs to interacting proteins (Table 1). PAR-CLIP has been applied to identify the transcriptome-wide binding sites of several RBPs and microRNA-containing ribonucleoprotein complexes.

Recently, researchers have elaborated on the methods for pulling down RNA-proteins complexes via antisense oligos that are complementary to targeted RNA sequences. Therefore, this type of capture is not limited by the requirement of antibodies against the proteins of interest for immunoprecipitation. In 2015, three groups established RNA-centric capture methods, including iDRiP-MS *(i*dentification of *d*irect *R*NA *i*nteracting *p*roteins), RAP-MS (RNA antisense purification coupled with mass spectrometry) and ChIRP-MS (comprehensive identification of RNA-binding proteins by mass spectrometry) to determine Xist RNA interacting proteins [18–20] (Table 1 and Fig. 4). In these three studies, cells were initially crosslinked to preserve RNA-protein interactions, and RNA-protein complexes were further purified by biotinylated antisense oligos along with highly denaturing purification conditions. ChIRP-MS utilizes formaldehyde to crosslink RNA-protein complexes, whereas RAP-MS and iDRiP-MS apply UV light for crosslinking (Table 1 and Fig. 4). Given that UV light is a short-range crosslinker, the RNA-protein interactions revealed by RAP-MS and iDRiP-MS tend to be direct. In contrast, the formaldehyde crosslinking fixes much larger macromolecular networks and generally leads to identify both direct and indirect factors.

To identify all RNA-binding proteins (RBPs), RBR-ID (proteomic identification of RNA-binding regions) introduces 4SU (4-thiouridine) into RNAs, crosslinks RNA-proteins by UV light, and compares the mass spectrometry of 4SU treated and non-treated samples [21]. An RNA-crosslinked peptide has a different mass, so the intensity of the signal is generally lower in the crosslinked sample compared to the non-crosslinked sample (Table 1 and Fig. 4). Using RBR-ID, about 800 previously unknown and known RNA-binding proteins, such as chromatin factors (CTCF, ATRX, HDAC1, DNMT3, EZH2, TET1, TET2 and HP1), were identified as RBPs in mouse embryonic stem cells [21].

### Methods for RNA-RNA interactions

**Fig. 5:**
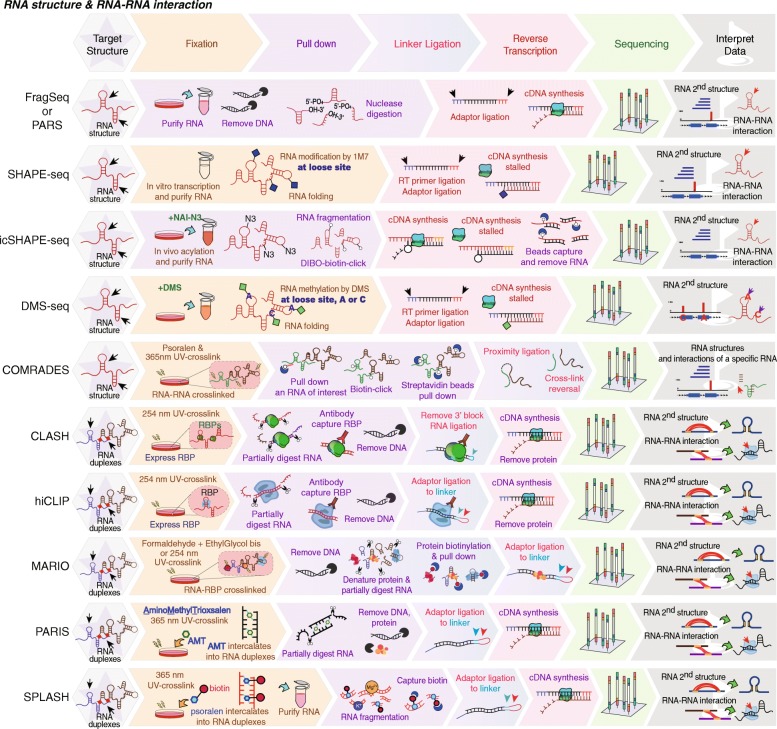
Profiling RNA structure and RNA-RNA interactions. FragSeq, PARS and SHAPE-seq reveal in vitro RNA structures by nuclease or chemical probing. DMS-seq, icSHAPE-seq, and COMRADES map in vivo RNA structures by modifying RNA with chemicals. CLASH and hiCLIP can identify RNA-RNA duplexes bound by a specific protein. MARIO, PARIS, and SPLASH map all RNA-RNA interactions by introducing a linker to ligate RNA and RNA

Unlike DNA, RNA is a single strand of nucleic acids and is capable of folding into 3D structures that range from simple helical elements to complex tertiary structures and quaternary ribonucleoprotein assemblies [85]. The changes in RNA structures directly affect their functions in response to cellular conditions. Recently, high-throughput techniques that combine nuclease digestion [22, 23, 86] or chemical probing [24–26, 28] with next-generation sequencing have revealed the single-stranded or double-stranded regions of RNA molecules. FragSeq (fragmentation sequencing) [22] utilizes nuclease P1, which specifically cleaves single-stranded nucleic acids (Table 1 and Fig. 5). PARS (parallel analysis of RNA structure) [23] maps RNA structure using RNase V1 or S1 nuclease to digest RNA to determine the double or single-stranded regions, respectively. In SHAPE-Seq (selective 2′-hydroxyl acylation analyzed by primer extension sequencing) [24–26], RNAs are treated with chemical probes (such as 1 M7) that covalently modify the RNA in loops and bulges, thus blocking reverse transcription at the modified sites. However, these methods may not represent the RNA structures in vivo due to applying in vitro transcription and in vitro folding of RNAs.

To profile genome-wide RNA structures in vivo, Ding et al. developed a method, called DMS-seq [28] (Table 1 and Fig. 5). In the DMS-seq, cells are treated with dimethyl sulfate (DMS) that methylates unprotected adenines (As) and cytosines (Cs) of RNA, resulting in the reverse transcriptase stalling at one nucleotide before DMS-modified As and Cs during cDNA synthesis. A year later, Chang’s group developed a method, termed icSHAPE [27] (in vivo click selective 2′-hydroxyl acylation and profiling experiment and NAI-N_3_) to probe RNA secondary structures in living cells for all four bases. They used a cell-permeable SHAPE reagent, NAI-N_3_, which adds an azide group to NAI (2-methylnicotinic acid imidazolide) to label flexible regions of RNA. After RNA isolation, a biotin moiety is selectively added to NAI-N_3_-modified RNA by copper-free click chemistry, allowing the biotin-streptavidin purification of modified RNA after RNA fragmentation. By comparison of in vivo and in vitro icSHAPE, they observed that all RNAs are less folded in vivo*,* suggesting that RNA structures largely depend on intracellular environments. Moreover, they found that regulatory RNAs, such as lncRNAs and primary microRNA (miRNA) precursors, preserve their structures better than mRNAs in vivo. To selectively enrich some specific RNA molecules, COMRADES (cross-linking of matched RNAs and deep sequencing) combines RNA capture and CLICK chemistry to probe RNA structures and RNA-RNA interactions [29]. In the COMRADES method, cells are crosslinked by azide-modified psoralen, following RNA capture using biotinylated probes, and a copper-free click-chemistry reaction is carried out to link a biotin moiety to the cross-linked regions, allowing the second selection of the cross-linked regions for sequencing (Table 1 and Fig. 5).

In addition, an RNA molecule can base-pair with other RNA molecules to form RNA duplexes (such as miRNA and its target RNA) bound by RBPs. To identify miRNA targets, Tollervey et al. developed a method, named CLASH (crosslinking, ligation, and sequencing of hybrids) [30, 31] to identify human AGO1 interacting RNA duplexes (Table 1 and Fig. 5). In the CLASH method, RNA-protein complexes are UV-crosslinked and purified by antibodies against RNA binding proteins, following the ligation of RNA-RNA hybrids, and the chimeric RNAs of RNA-RNA duplexes are deep sequenced. Similarly, hiCLIP [32] also can map RNA-RNA duplexes bound by RBPs. In the hiCLIP method, a linker-adapter is introduced to the ligation step of RNA-RNA duplexes to improve the ligation efficiency (Table 1 and Fig. 5). Moreover, the ligation of two chimeric RNAs has been applied to other methods including MARIO (Mapping RNA interactome in vivo) [33], PARIS [34] and SPLASH [35], which map all RNA-RNA interactions (RNA structures and interactome) in living cells.

### R-loops in gene regulation and genomic instability

R-loops are three-stranded nucleic acid structures in which a strand of RNA hybridizes with a strand of DNA, while the other strand of DNA loops out. The R-loop structure was first described in 1976 in the study [87] in which Thomas et al. showed that RNA could hybridize to double-stranded DNA in the presence of 70% formamide in vitro. A year later, Roberts and Sharp used R-loop hybridization technique to map an adenovirus 2 (Ad2) mRNA to its genome and found that some DNA sequences of the Ad2 coding gene were not hybridized with the matured RNA, suggesting that Ad2 genome consists of non-coding sequences, later known as introns [88, 89]. Recently, genome-wide studies have shown that R-loops are found in vivo, especially enriched in promoter regions [36]. Ginno et al. demonstrated that R-loop formation is involved in gene regulation via its potential to protect DNA from methylation [36]. Moreover, recent reports showed that antisense long noncoding TARID (TCF21 antisense RNA inducing promoter demethylation) forms an R-loop at the *TCF21* promoter, thus facilitating GADD45A binding, local DNA demethylation and *TCF21* expression [90, 91]. Another example is GATA3-AS1 lncRNA*,* which forms an R-loop structure with the central intron of *GATA3-AS1* and tethers the MLL H3K4 methyltransferase to *GATA3* gene locus, thereby regulating *GATA3* expression [92]. Furthermore, it has been proposed recently that R-loops act as intrinsic Pol II promoters to induce lncRNA transcription [93]. The depletion of R-loops by overexpressing RNase H1 causes the selective reduction of antisense lncRNA transcription [93].

It is generally believed that R-loops form *in cis* during transcription when a nascent RNA hybridizes to the DNA template behind the moving RNA polymerase [94]. However, research in yeast suggests that RNA:DNA hybrids can form *in trans*, which means that an RNA transcript at one locus hybridizes with homologous DNA at another locus [95], and the hybrids are likely involved in homologous recombination. Moreover, excellent studies have shown that genome instability arises from lesions generated from the formation of R-loops [95, 96]. Because transcription and replication share a common DNA template, when replication forks encounter transcription machinery, it results in transcription-replication collisions that lead to DNA damage. Therefore, the persistent RNA:DNA hybrids could be threats for genomic rearrangements [96].

### Methods for mapping R-loops

**Fig. 6:**
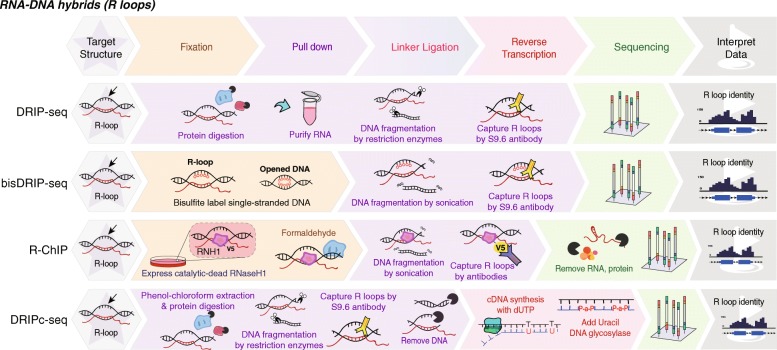
Profiling R-loops (RNA-DNA hybrids). DRIP-seq and bisDRIP-seq utilize S9.6 antibodies to pull down DNA:RNA hybrids (R-loops), and the captured DNA fragments are sequenced. DRIPc-seq builds on DRIP-seq, and the captured RNA fragments are reversed transcribed and sequenced. R-ChIP captures R-loops using a standard ChIP-seq protocol to pull down R-loop/catalytic-dead RNase H complex

To map R-loops in a genome-wide scale, Ginno et al. developed a method, named DRIP-seq (DNA-RNA immunoprecipitation coupled to high-throughput sequencing), which utilizes an antibody (S9.6) [36] to specifically purify RNA:DNA hybrids (Table 1 and Fig. 6), and the captured DNA fragments are further sequenced. Conventional DRIP-seq generally produces a robust signal but has a limited (approximately kilobase) resolution, a higher background, and a lack of strand specificity. Another method, named DRIPc-seq (DNA:RNA immunoprecipitation followed by cDNA conversion coupled to high-throughput sequencing), which builds on DRIP, except that a strand-specific RNA sequencing is performed to profile R-loops [40]. DRIPc-seq increases the resolution of R-loop profiling and shows the strand-specificity. However, the sensitivity of DRIP for revealing authentic R-loops in vivo has been doubted due to the fact that the immunoprecipitation for R-loops is usually performed using isolated genomic DNA without any crosslinking. Thus R-loops could be disrupted or formed in vitro after cell lysis. Dumelie et al. developed a method, named bisDRIP-seq (bisulfite-based DRIP-seq) [38], which selectively labels the single-stranded DNA that loops out from an R-loop. They used bisulfite to convert cytosine residues into uracil residues within the genomic DNA regions that contain single-stranded DNA simultaneously when cells were lysed. Their results showed that bisDRIP-seq could map R-loops at near nucleotide resolution and detect the boundaries of R-loops. In contrast to DRIP, R-ChIP (RNase H chromatin immunoprecipitation) utilizes an RNase H, which specifically recognizes DNA:RNA hybrids in vivo to map R-loops [39]. By expressing a catalytically dead enzyme, R-ChIP captures R-loops using a standard ChIP-seq protocol, which involves both fixation to stabilize R-loop/RNase H complex and sonication to increase the resolution (Table 1 and Fig. 6).

### lncRNAs mediate phase separation of membrane-less organelles

**Fig. 7:**
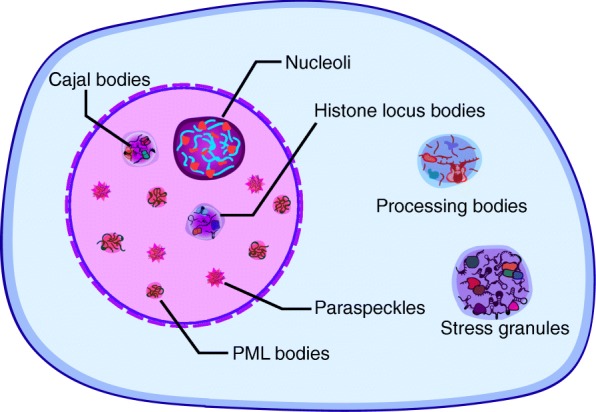
RNA-protein droplets in cells. RNA granules are made out of protein and RNA complexes, and their formations are driven by liquid-liquid phase separation. Nucleoli, paraspeckles, Cajal bodies (CBs), histone locus bodies (HLBs) and promyelocytic leukemia (PML) bodies are RNA assemblies in the nucleus. P-bodies and stress granules are found in the cytoplasm. RNAs act as a regulatory element to control their sizes and properties. The imbalance of RNA/protein ratio in such RNA assemblies could lead to human diseases

Cellular organelles such as mitochondria and Golgi apparatuses composed of lipid bilayer membrane structures, which help the formation of compartments and separate biological processes within a cell. In contrast, membraneless structures are formed through a process known as liquid-liquid phase separation (LLPS) and are made of RNA-protein droplets [97]. One of the most prominent membraneless structures in the nucleus is the nucleolus [98], which produces the ribosomal RNA and consists of a variety of proteins and RNA. Studies have shown that rRNA transcription is important for nucleolar assembly [50, 51, 99, 100]. Other membraneless structures, such as paraspeckles, Cajal bodies (CBs), histone locus bodies (HLBs) and promyelocytic leukemia (PML) bodies are also found in the nucleoplasm as phase separated-like droplets (Fig. 7), while stress bodies and process bodies are RNA granules in the cytoplasm. DNA is typically absent from the interior of these liquid-like droplets, whereas lncRNAs serve as scaffolds for their formation and maintenance [101–103].

How do RNAs promote phase separation synergistically with protein-protein interactions? The in vitro studies reported that RNA repeats, such as trinucleotide repeat and G-quadruplex, can undergo a phase transition to form either a condensed liquid or a gel-like state [104] through the multivalent base-pairing between RNA molecules. Droplet-like assemblies of RNA are associated with certain neurodegenerative diseases, including Huntington disease, muscular dystrophy, and amyotrophic lateral sclerosis [105]. It has been proposed that such gel-like RNA foci may contribute to neuronal dysfunction in vivo. A recent study showed that RNA plays an important role in the phase behavior of prion-like RBPs, such as TDP43 and FUS [106], which are largely soluble in the nucleus but form solid pathological aggregates when mislocalized to the cytoplasm. The in vitro studies indicated that the ratio of RNA/protein is important for droplet formation and phase separation [106]. Remarkably, reduction of nuclear RNAs or disruption of RNA binding leads to excessive phase separation and the formation of cytosolic assemblies in cells [106], suggesting that the higher RNA concentrations in the nucleus act as a buffer to prevent aggregation of RBPs in the cytoplasm. Then the question is how the RNA/protein ratio is regulated in such droplets. Hondele et al. recently reported that RNA flux into and out of phase-separated organelles is controlled by RNA-dependent DEAD-box ATPases (DDXs), which contain low-complexity domains (LCDs) that are crucial for the formation of multivalent meshworks within membraneless droplets [107]. In addition, Ries et al. showed that methylation on mRNAs triggers phase separation of endogenous compartments, such as P-bodies, stress granules or neuronal RNA granules [108]. These studies indicate that the abundance of nuclear RNAs and the modifications of RNA contribute to the dynamics of such membraneless organelles. However, there are intriguing questions that remain elusive. How do RNA structures impact on phase separation? How do RNA-mediated droplets involve in chromatin organization and gene regulation? What signals trigger the reorganization of such structures?

## Conclusions

The Human Genome and ENCODE Projects have shown that above 90% of the genome is transcribed [1, 72] into non-coding RNAs. However, the functions of most non-coding RNAs remain largely unknown. In the last decade, emerging new techniques combined with high throughput sequencing have profiled the interactions between DNA, RNA, and proteins, thus facilitating the studies of lncRNAs functions in cells. LncRNAs can act as a guide, or decoy, or a scaffold for protein complexes to mediate the epigenetic regulation [109]. Chromatin-associated lncRNAs involves nuclear architecture and chromatin conformation. Given that lncRNAs are long and mobile, they could serve as bridges to mediate chromatin looping and to drive the inter- or intra-chromosomal interactions [44, 45]. Moreover, RNA is able to hybridize with DNA to form R-loops, which have been found to contribute to gene regulation and genomic instability. RNA also mediates the liquid-liquid phase separation through its ability being as a multivalent scaffold for the binding of RBPs, thus regulating the sizes and the dynamics of membraneless organelles that carry biological processes. It is still a beginning to uncover the surface of the lncRNA world. There are still a lot we don’t know and plenty of work that needs to be done. Because RNA molecules consist of specific sequences, it is realistic and easy to design drug targets by blocking their actions using antisense oligos, RNA interferences, or aptamers. We hope that by understanding the mechanisms of lncRNAs action, RNA-centric therapies could be potential options to treat human diseases.

## Data Availability

Not applicable.

## Abbreviations

lncRNAs: Long non-coding RNAs
R-loops: RNA:DNA hybrids
CTCF: CCCTC-binding transcription factor
TADs: Topologically associated domains
ChIP: Chromatin Immunoprecipitation
eRNAs: Enhancer RNAs
RBPs: RNA binding proteins
4SU: 4-thiouridine
IGF2: Insulin-like growth factor 2
ATRX: Alpha Thalassemia/Mental Retardation Syndrome X-Linked
HDAC1: Histone Deacetylase 1
DNMT3: DNA methyltransferase 3
EZH2: Enhancer Of Zeste Homolog 2
TET1: Ten-eleven translocation *methylcytosine dioxygenase* 1
TET2: Ten-eleven translocation *methylcytosine dioxygenase* 2
HP1: Heterochromatin Protein 1
PRC1: Polycomb Repressive Complex 1
PRC2: Polycomb Repressive Complex 2
*AGO1*: Argonaute RISC Component 1
1 M7: 1-methyl-7-nitroisatoic anhydride
GADD45A: Growth arrest and DNA damage inducible alpha
GATA3: GATA binding protein 3
